# Natural products as promising modulators of breast cancer immunotherapy

**DOI:** 10.3389/fimmu.2024.1410300

**Published:** 2024-07-10

**Authors:** Aljawharah Alqathama

**Affiliations:** Department of Pharmaceutical Sciences, Faculty of Pharmacy, Umm Al-Qura University, Makkah, Saudi Arabia

**Keywords:** breast cancer, immunotherapy, checkpoints, immune cells, natural products

## Abstract

Breast cancer (BC) is the most common malignancy among women and is considered a major global health challenge worldwide due to its high incidence and mortality rates. Treatment strategies for BC is wide-ranging and include surgery, radiotherapy, chemotherapy, targeted hormonal therapy and immunotherapy. Immunotherapy has gained popularity recently and is often integrated as a component of personalized cancer care because it aims to strengthen the immune system and enable it to recognize and eradicate transformed cells. It has fewer side-effects and lower toxicity than other treatment strategies, such as chemotherapy. Many natural products are being investigated for a wide range of therapeutic pharmacological properties, such as immune system modulation and activity against infection, auto-immune disease, and cancer. This review presents an overview of the major immune response-related pathways in BC, followed by detailed explanation of how natural compounds can act as immunomodulatory agents against biomolecular targets. Research has been carried out on many forms of natural products, including extracts, isolated entities, synthetic derivatives, nanoparticles, and combinations of natural compounds. Findings have shown significant regulatory effects on immune cells and immune cytokines that lead to immunogenic cancer cell death, as well as upregulation of macrophages and CD+8 T cells, and increased natural killer cell and dendritic cell activity. Natural products have also been found to inhibit some immuno-suppressive cells such as Treg and myeloid-derived suppressor cells, and to decrease immunosuppressive factors such as TGF-β and IL-10. Also, some natural compounds have been found to target and hinder immune checkpoints such as PD-L1.

## 1 Introduction

Breast cancer is thought to be the second most frequent cause of cancer-related deaths in women worldwide. It has been estimated that one in three women may be at risk of developing BC in their lifetime and it has also been predicted that by 2050 there will be 3.2 million new cases worldwide (1). Cancer research data estimated that 287,850 women in the USA would be diagnosed with BC in 2022, and that 43,250 women would die from the disease. These statistics place BC second only to lung cancer as the leading cause of women’s cancer deaths (2). BC is a heterogeneous disease and classified into four categories based on the immunohistochemical expression of hormone receptors, with different behavior and responsiveness to treatment and clinical results (1). The categories are as follows. Group 1 (luminal A), which is characterized by estrogen receptor (ER) positive and progesterone receptor (PR) positive; Group 2 (luminal B) shows ER positive, PR negative, and human epidermal growth factor receptor positive (HER2+). The third group is HER2+ positive only, and Group 4 (basal-like), known as triple-negative (TNBC), lacks the expression of any of the above receptors (1, 3).

## 2 BC treatment

For breast cancer treatment, choice of strategy is based on the grade, stage, and BC molecular subtype in order to achieve optimal therapy outcomes. Surgery is the most standard approach for BC treatment in the form of mastectomy, either by total excision of the breast, or by lumpectomy in which breast-conserving surgery removes the cancerous tissue in part of the breast. In addition, radiotherapy, chemotherapy in both neoadjuvant and adjuvant chemotherapy forms, hormone therapy, and targeted therapy are the mainstream options for BC treatment (4).

Most conventional anticancer drugs kill tumor cells directly by interfering with basic cell functions to the degree that cells are no longer able to survive (4). Recent research has focused on the crucial role of the immune system in fighting cancer cells, leading to the emergence of immunotherapy to provide a transformative new method of comprehensive cancer treatment. Moreover, immunotherapy builds on the natural ability of the immune system to recognize and eradicate non-normal cells through the process of tumor immune surveillance. This involves a dynamic and orchestrated interplay of innate and acquired immune responses. Tumor cells avoid immune surveillance by suppressing the body’s immune system in various ways, thus enabling tumor immune escape. To counteract this, combinations of immuno- and chemotherapies are being developed to strengthen immune system response to tumors and to minimize the negative effects of chemotherapy (4, 5).

## 3 Role of the immune system in cancer

The immune system is highly complex and consists of collections of cells, chemicals, and organs, such as the skin, lungs, and gastrointestinal tract, which protect the major organs and other areas from foreign antigens. The immune system is activated as a defense mechanism when it detects abnormalities, for example, underactivity, that could allow microbial infection (i.e. by bacteria, fungi, or parasites), or overactivity, that could lead to allergy or autoimmune disease. Major components of the immune system are immune cells of different types, such as lymphocytes, dendritic cells (DCs), monocytes/macrophages, and natural killer T cells (NKs) (6). The tumor microenvironment (TME) is composed of both cancerous and non-cancerous cells (such as immune cells, endothelial cells, and fat cells), which play a major role in transforming normal cells into tumor cells. As a large proportion of the TME consists of immune cells, they play an essential role in controlling pro- and anti-tumor immune responses; thus the characteristics of the TME are intrinsically related to the efficacy of immunotherapy (7).

In this review we gather together findings from recent *in vitro*, *in vivo* and clinical studies that evaluate the effects of various natural products used as immunotherapeutic agents on BC models. We first provide detailed insight into the role of the TME, immune cells, cytokines, and immune checkpoints in BC immune tolerance. This is followed by a detailed discussion of research evidence for the efficacy of natural products in triggering or improving immunogenic activity in different multi-cell and multi-biomolecular pathways, and in inhibiting or eradicating tumor cells. To conclude, we present a summary of the advantages and disadvantages of natural products as immunotherapeutic agents in BC.

## 4 Tumor immune microenvironment and breast cancer

Detailed knowledge of the TME is essential to the development of potential immunotherapeutic strategies, since its composition and characteristics strongly influence the genesis and progression of tumors (8). In BC, the TME is composed of either cellular, soluble, or physical components. Cellular types is classified into local (intratumoral), regional (breast) and metastatic categories. Local cells of the TME include cancer cells as well as unfiltered inflammatory cells such as macrophages, lymphocytes, DCs, and neutrophils. The interaction between cancer cells and adjacent stromal cells, including stromal fibroblasts, vascular/lymphatic endothelial cells, adipocytes and endothelial, fall into the regional category, while metastatic cells include host cells at metastatic areas, e.g. lymph nodes and distant organs, that form new TMEs (9).

### 4.1 Immunosuppressive/immunostimulant cells and factors

The TME contains two major classes of immune cells: immuno-suppressive and immuno-stimulating. Their activity is dependent on innate and adaptive immune system responses (10). Several types of immune cells are active in cancer cell escape immune editing, such as tumor infiltrating lymphocytes (TILs). TILs have different functions and transcription factors, and occur as several subtypes, including Th cells (helper cells), CTLs (cytotoxic CD8+ T lymphocytes), and Tregs (regulatory T-cells). Other cells with a role in immune regulation are CD4+ effector T cells, tumor-associated macrophages (TAMs), myeloid-derived suppressor cells (MDSCs), DCs, NKs, and mast cells (11). Several immunosuppressive factors, such as prostaglandin E2 (PGE2), interleukin-10 (IL-10), and transforming growth factor-β (TGF-β), are secreted by these cells to regulate the immunosuppressive network (5).

Studies have shown a high number of TILs in BC, which are composed mainly of T cells of different classes, and fewer B cells (9). The adaptive immune response is supported by CD4+ T cells and CD3+ T cells, as well as CTLs. When APCs (antigen-presenting cells) such as macrophages and DCs detect tumor antigens, CTLs release granzyme and perforin, mediated by interferon-γ (IFN-γ) secretion, to eliminate cancer cells. In addition, IFN-γ and IL-12 signaling activate CD4+ T-cells, which are type 1 helper (Th1) cells, allowing APCs to license the differentiation of CD8 T-cells and clonal expansion. Th1 cell presence is associated with better clinical outcomes in BC patients as these cells activate CD8+ T lymphocytes, triggering their cytotoxic activity by freeing pro-inflammatory cytokines (9). Other T helper cells, Th2 and Th17, play a role in BC progression as well as follicular helper T-cells, which largely regulate the maturation of antigen specific B cells, enhancing local memory and elevating the development of tertiary lymphoid organs, leading to an immune response that targets local tumors (12). Major regulators of immune system homeostasis are Tregs, as their existence in the TME elevates immune system capability to act as an immunosuppressive through direct cell-cell contact suppression and immunosuppressive cytokines (IL-10, TGF-β) (13). Tregs, which express Foxp3, belong to a class of suppressive cells and act to suppress effector T cells, preventing immune-mediated rejection of tumors (14).

NKs are a class of APC which play a major role in immune tolerance in BC. They are cytotoxic innate lymphocytes which act to lysate and eradicate malignant cells, and this eradicating mechanism is independent of major histocompatibility complex I (MHC-I) molecules and antibodies. In order to avoid tumor-invading cytotoxic T lymphocytes detecting the presence of tumor cells, MHC-I expression on the surface of the tumor cell is often inhibited or eliminated. NK cell inhibitory receptors are able to detect this lack of MHC-I, and the immunogenic effect of NK cells is evident in their contribution to regulating the function of multiple immune cells, including DCs, macrophages, and T and B cells (15). Principal components of the TME are DCs, which are thought to be one of the most potent types of APC, and present antigens, including tumor-derived antigens, to T-cells. DCs have two major phenotypes with different surface protein expression, known as myeloid and plasmacytoid populations. DCs become mature and stimulate the immune system by interacting with T cells. Cancer cells, however, have the ability to inhibit the maturation of DCs, which results in tumor-infiltrating DCs having an underdeveloped phenotype. Tumor-derived antigen cross-presentation is therefore inhibited, co-stimulatory molecules show downregulation, so DC antitumor functions can be impaired (9).

Findings show that tumor-infiltrating B cells could have both pro-tumor and anti-tumor effects, which greatly depend on the components of the TME and BC phenotypes. Moreover, tumor-specific antigen recognition, antibody production, and APC functioning have all been shown to affect the anti-tumor properties of B cells (9). On the other hand, B cells have been shown to mediate tumor growth, as regulatory B cells express inhibitory molecules such as programmed cell death-ligand 1 (PD-L1) and FAS ligands, as well as anti-inflammatory mediators such as TGF-β, IL-10, and IL-35, resulting in the suppression of immune responses leading to cancer cell immune escape (16).

One of the main innate immune cells in BC are macrophages, which occur as two polarized phenotypes, the M1 alternative macrophages and the M2-like TAMs (9). M1 macrophages are activated by IFN-γ and tumor necrosis factor-alpha (TNF-α), which are released by Th1 cells, thus causing production of reactive oxygen species and release of pro-inflammatory cytokines (IL-12 and IFN-γ), processes which, in turn, stimulate anti-tumor activity (17). The other activated macrophage phenotype, M2-like TAMs, are switched on by Th2 cells cytokines such as IL-13, IL-10 and IL-4, leading to tumor progression, inducing angiogenesis and metastasis, and inhibiting the anti-tumor response (18). The development of the M2-like TAM macrophage is encouraged by the existence of IL-4 and IL-13 in the TME. Tumor cells can produce macrophage-derived chemokines such as C–C motif chemokine 22, which then bring monocytes into the tumors; if immuno-suppressive conditions prevail, these monocytes will then differentiate into TAMs (5). Immune cells called neutrophils also have the potential to promote or hinder tumor development. As tumor-associated neutrophils (TANs) they function as tumor-infiltrating immune cells. They exhibit different phenotypes (N1 and N2) in which N1 cells have the pro-inflammatory and anti-tumor properties of TANs, triggered by IFN-γ and IFN-β exposure (9). N2 neutrophils are triggered by TGF-β exposure, producing anti-inflammatory and pro-tumor TANs (18).

MDSCs are known as immunosuppressive populations and are characterized into two phenotypes; polymorphonuclear or granulocytic MDSCs and monocytic MDSCs. These populations are precursors of bone marrow and have the ability to suppress the immune response via the repression of CTLs and NKs and the secretion of immunosuppressive cytokines including IL-10 and TGF-β, as well as the induction of expression of PD-L1 (19). Mast cells promote the growth of tumor cells via the secretion of H+, NO, chondroitin sulfate, and oxidized polyamines, leading to the induction of the non-degranulated mode of mast cells, resulting in an immunosuppressive effect. Moreover, the cytokines secreted by mast cells, such as histamine, IL-10, and TGF-β result in the suppression of effector T cells, and the secretion of PGE2 affects the migration and function of DCs, all of which strengthens immunosuppression within tumors (5).

Recent research suggests that certain types of human immune cells exhibit this kind of two-way oppositional mechanism in BC occurrence, that is, alterations in cell composition mean they are able either to encourage tumor growth or suppress it (20). In breast cancer, TILs can affect cancer cells and immune cells in different ways, depending on the stage of the cancer and the specific cell phenotype. This means either a pro- or anti-tumor response, leading either to the promotion of tumor cell proliferation and spread, or its suppression and destruction (21).

Studies in the BC microenvironment show that TILs can affect the response of cancer cells by either promoting tumor generation, or triggering suppression and apoptosis. Both CD4+ and CD8+ T cells can affect the adaptive immune response; however, when activated, CD8+ differentiates into CTLs, while CD4+ cells divide into sub-populations of T helper cells (Th1, Th2, Th17) (20). The CD8+ cell types cause tumor cell destruction and are dominant in the BC microenvironment, whereas CD4+ subpopulations can produce either pro- or anti-tumor activity. For example, Th1 CD4+ cells secrete pro-inflammatory mediators INF-γ and TNF-α, and trigger the anti-tumor activity of NKs, thus activating a powerful anti-cancer immune response (11, 20). In a contradictory fashion, the cell subtype Th2 CD4+ instead promotes tumorgenicity and encourages metastasis by releasing cytokines IL-4, IL-5, and IL-13, but it also releases IL-10, which can influence either the growth or destruction of cancer cells. Another subtype, Th17 CD4+, releases TGF-β, which is known to encourage cancer cell progression (20). Similarly, different phenotypes of TAMs may polarize to either M1-like or M2-like macrophages, which are linked with tumor progression and suppression respectively (22). The M1 phenotype promotes the apoptosis of cancer cells via CTL recruitment and activation of the adaptive immune responses, while M2 attracts Th2 and Treg cells, encouraging cancer cell growth, tissue remodeling and tumor angiogenesis (23). Other clinical research supports findings that M2-like macrophages promote BC cancer cell proliferation and overall negative outcomes (22).

### 4.2 Immunological checkpoints

Literature has shown that certain regulatory molecules play a physiological role in the suppression of self-immune responses. When these regulatory molecules, known as immune checkpoints, become dysregulated, they cause evasion of immune-mediated destruction. In pathological conditions such as BC, they include programmed cell death 1 (PD-1), cytotoxic T lymphocyte-associated protein 4 (CTLA-4) and its ligands (PD-L1/2) and T-cell immunoglobulin and mucin domain containing protein-3 (TIM-3) (24). Targeting immune checkpoints with immune checkpoint inhibitors (ICIs) has brought advances in in cancer immunotherapy because ICIs accelerate the anti-cancer immune response to eradicate malignant tumors. ICIs can restore the suppressed immune cells as a recognizers of cancer cells by blocking immune checkpoint interference in the cross-talk between immune cells and cancer cells, thus reactivating natural immune responses in BC patients. Current clinical practice using ICI treatment shows dramatic improvement in survival rates in advanced-stage and metastatic cancers (25).

## 5 Natural products as immunotherapy agents in BC

Humankind has used natural products to treat disease for at least three thousand years (26). The pharmacologically active constituents of many different plant, animal, marine, and microbial organisms continue to provide a wide range of molecules that produce healing responses in the body (27). Many recent studies have demonstrated the potential anti-cancer activities of naturally-sourced compounds, leading to the development of new anti-cancer agents (28). Moreover, around 47% of current anti-cancer drugs are derived from natural compounds (5). The chemical diversity of natural products is reflected in their different effects on cancer cells, including cell proliferation inhibition, apoptosis induction, suppression of metastasis and angiogenesis, autophagy modulation and reversal of multidrug resistance. They are also able to manipulate the tumor microenvironment or fine-tune it to bring about immune response regulation to eradicate tumor cells (7). Methods of treating cancer with immunotherapy, such as ICI treatment, adoptive T cell transfer therapy, and cancer vaccination, have all been successfully combined with anti-cancer agents derived from natural products to improve treatment efficacy (26). Research into the beneficial effects of natural products in anti-cancer and immunomodulatory treatment suggests that many are valuable candidates for adjuvant use in tumor immunotherapy (5).

**Figure 1:**
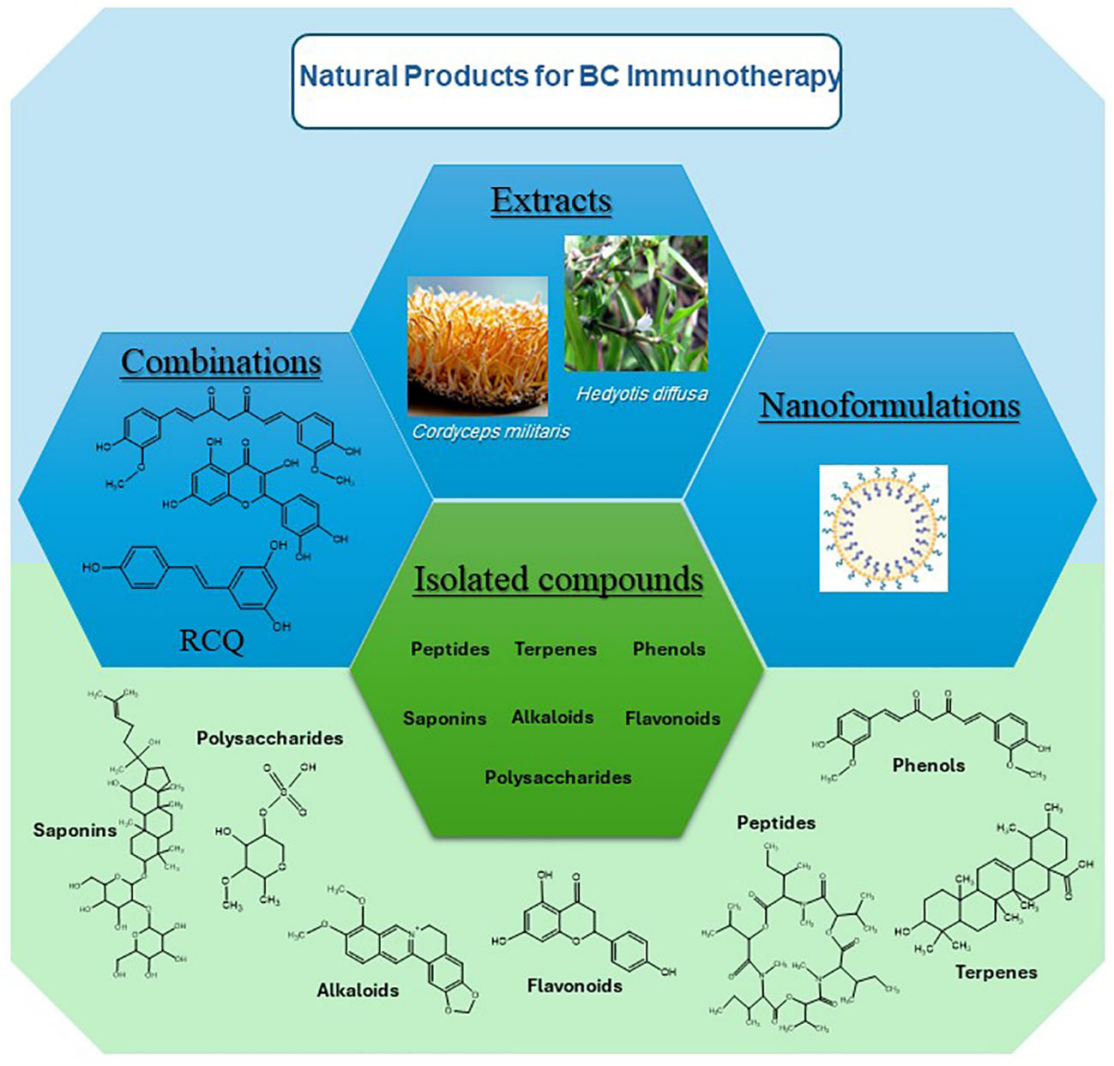
Different sources and chemical classes of immunotherapeutic natural products on BC.

**Figure 2:**
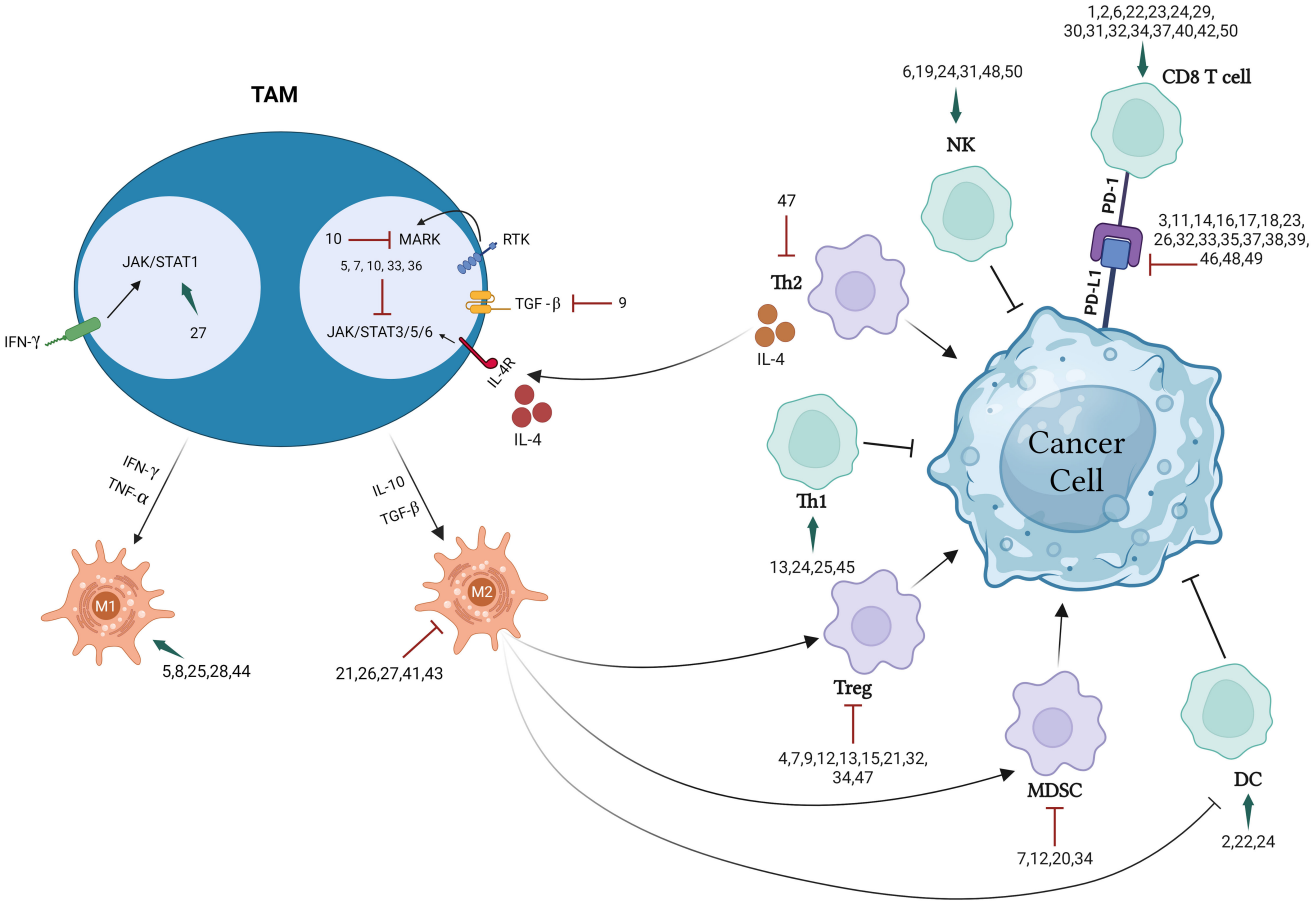
Natural products regulate biomolecular pathways to restore immunogenic tumor-cell death.

This extensive review presents and discusses research findings on 50 preparations including isolated entities, extracts, several combination and nanoparticle formulations, all of which have significant immunotherapeutic potential in targeting the TME, immune cells, cytokines, and immune checkpoints. Different chemical classes which have been found to modulate immune response include alkaloids, flavonoids, terpenes, phenolics, and peptides as illustrated in **Figure 1** below. **Figure 2** illustrate the biomolecular mechanisms that are dysregulated in BC and how natural products restore theses pathways to induce immunogenic tumor-cell death.

### 5.1 Phenolic compounds

Curcumin (1) is known as the chief constituent of the culinary spice turmeric. It demonstrates important biological properties, including anti-inflammatory, antimicrobial and anticancer activity. It can regulate immune response in BC as reported by Krishnamurthy et al., where curcumin and mannan, a component of the Aloe vera plant, inhibited the proliferative activity of immune cells, including peripheral blood mononuclear cells (PBMCs) such as lymphocytes, monocytes and macrophages (29). In some conditions the effects of curcumin are limited due to solubility issues, as it has poor solubility in neutral or acidic media and is unstable in alkaline conditions (30). However, delivery of curcumin to the target site in a sustainable and controlled manner can be achieved through nanotechnology, such as curcumin-loaded polymeric nanoparticles (2) and a nano-vaccine containing cytosine-phosphate-guanine and antigenic peptides. Injection of this formula in a BC model triggered immunogenic cell death of cancer cells and activation of DCs. DCs stimulation significantly improved tumor-specific CD8+ T-cell responses resulting in tumor inhibition (**Table 1**) (31).

**Table 1:** List of natural products with immuno-tumor therapeutic effects on breast cancer model.

Natural Compound	Category	Immunomodulatory effect in BC	Reference
Curcumin (1)	Phenolic compounds	Inhibition of proliferative activity of some immune cells like PBMC and trigger Th2 activity	(29)
Curcumin nanoparticles (2)		Stimulation of DCs and tumor-specific CD8+ T-cells	(31)
Resveratrol (3)		Inhibition of PD-L1, restore T-cell function	(32)
HS-1793 (4)		Change subpopulations of tumor-derived T cells including the CD25+ and Treg population	(33)
Vanillic acid (5)		Promotion of the polarization of macrophages to a M1 phenotype	(34)
XK-81 (6)		Elevation of CD8+ T and NKs, modulation the ratio of M1/M2 macrophage and elevation of immune-related cytokines, including TNF-α, IL-1β, and IL-12	(35)
Ursolic acid (7)	Terpenes	Modulation of CD25+ Foxp3+ T cells via the inhibition of STAT5 phosphorylation, IL-10 secretion, reduced MDSC and Tregs	(36)
Anemoside A3 (8)		Shifting M2-TAM to M1-TAM phenotype and inhibiting TAM- TNBC crosstalk	(37)
Oridonin (9)		Attenuation of Tregs cells and expression of TGF-β receptor	(38)
Crassolide (10)		Reduction of the level of CD24 on the surface of cancer cells and blocked mitogen-activated protein kinase 14 activation and STAT	(39)
Triptolide (11)		Down-regulation of PD-1/PD-L1 pathway	(40)
Naringenin (12)	Flavonoids	Reduction of infiltrating MDSCs and Treg cells, the upregulation of INF-γ and IL-2-releasing T cells and reducing the production of TGF-β1	(41, 42)
Naringenin with cryptotanshinone (13)		Enhancing Th1 cells, modulating Treg cells activity through JAK2/STAT3 pathway	(43)
Apigenin (14)		Inhibition of interferon-γ-induced PD-L1 expression	(44)
Salvigenin (15)		Reduction of IL-4 level, elevation of IFN-c with suppression CD25+Foxp3+ Treg cells	(45)
Myricetin (16)		Inhibition of PD-L1	(46)
Sativan (17)		Suppression of PD-L1, N-cadherin, snail and vimentin	(47)
Hesperidin (18)		Suppression on PD-L1 via inhibition of Akt and NF-κB signaling pathway	(48)
Berberine (19)		Elevation of infiltration of NKs	(49)
3,3′-Diindolylmethane (20)		Inhibition of MDSCs via downregulation STAT3 pathways and improving the therapeutic effect of PD-1 antibody	(50)
Prodigiosin (21)		Reduction of the number and differentiation of Tregs, inhibition of M2 polarisation and induction of TAM infiltration	(51)
Polyactin A (22)	Peptides	Stimulation of DCs, CD4+ and CD8+ T lymphocytes	(52)
Enniatin A (23)		Reduction of PD-L1 levels, promotion CD8+ T cell-dependent antitumor	(53)
Fucoidan (24)	Polysaccharides	Potentiation of CD8+ T cells, macrophages, DCs and NKs, elevation of pro-inflammatory mediators of Th1 and Tc1 cells (IFN-γ and TNF-α).	(54)
Nanoformulation of fucoidan with doxorubicin (25)		Shifting from M2 to M1 TAM phenotype polarization and increasing Th1 immune response	(55)
Combination of oligo-fucoidan and Olaparib (26)		Suppression of PD-L1 levels, subpopulations of CD44/CD24 and M2 macrophage, induction of antitumoral M1 macrophages	(56)
Lentinan (27)		Suppression of IL-4-induced M2 macrophage polarization and activation of JAK/STAT signaling pathway	(57)
Polysaccharide (SYQ) (28)		Induction of M2 to shift to anti-tumor M1 phenotypes	(58)
Polysaccharides GP (29)		Promotion of APC, cytotoxic T-lymphocytes and activated macrophages	(59)
Taccaoside A (30)	Saponins	Enhancement T lymphocyte (mTOR1)-Blimp-1 signal	(60)
Ginsenosides (31)		Increasing of NKs and memory T cell	(61)
Eribulin mesylate (32)	Miscellaneous	Reduction of PD-L1 and FOXP3	(62)
Sesamin (33)		Downregulation of PD-L1 expression through the suppression ERK JAK1/STAT signaling activity	(63)
Artemisinin (34)		Reduction of Tregs and MDSCs and increasing CD4+ IFN-γ+ T cells and cytotoxic T cells	(64)
Oleuropein (35)		Inhibition of PD-L1 expression	(65)
Salinomycin (36)		Restoring the proliferation of T cells and suppression of JAK/STAT pathway and IFN-γ-induced activation of the NF-κB pathway	(66)
Metformin (37)		Reduction of PD-L1 levels, activation of AMP-activated protein kinase activation and cytotoxic T cell activity	(67)
α-TOS nanoparticles (38)		Suppression of the expression of PD-L1 and modulation of cytotoxic lymphocytes	(68)
Neo-tanshinlactone (39)		Inhibition of PD-1/PD-L1 interaction	(69)
MZ58 (40)		Activation of CD8+ T cell	(69)
RCQ (41)		Shifting TAM cells to M2 TAMs and TANs to N2 TANs	(70)
Ethanolic extracts of *Cordyceps militaris* (42)	Extracts	Stimulation of proliferation of tumor-specific T cells, level of cytokines TNF-α, IL-1β and IL-6	(71)
XIAOPI extract formula (43)		Decreasing polarization and proliferation of M2-type macrophages	(37)
*Cordyceps sinensis*, *Taraxacum mongolicum, Coriolus versicolor* (44)		Shifting of M2-TAM to M1-TAM phenotype	(37)
*Coriolus versicolor* (45)		Activation of DCs	(72)
*Astragalus membranaceus* (46)		Reduction of the expression of PD-L1 via the Akt/mTOR/ribosomal protein S6 kinase beta-1 pathway	(73)
*Diospyros peregrina* (47)		Suppression of CD25+ Foxp3+Treg cells, reduction of release of Th2 cytokine (IL-10 and IL-4) and elevation of the release of Th1cytokines, including (IL-12 and IFN-γ).	(74)
*Sarcodon imbricatus* (48)		Decreasing the expression of PD-L1 and elevation of IL-2, IL-6 and TNF-α, and NKs activity	(75)
*Hedyotis diffusa* and *Scutellaria barbata* (49)		Reduction of the expression of PD-L1, β-catenin, and cyclin D1 causing inactivation of MAPK and Akt signaling pathways	(76)
Camel milk (50)		Elevation CD+4, CD+8, NKs	(77)

Resveratrol (3) belongs to the class of stilbenes, and is recommended for a number of different pathological conditions. Its immunological effect on BC has been tested, revealing that resveratrol inhibits glyco-PD-L1-processing enzymes (α-glucosidase/α-mannosidase) and PD-L1 dimerization and blocks the PD-1/PD-L1 interaction. Thus, it in the process increases cytotoxic T-lymphocyte activity and restores T-cell immune function in tumor tissue (32). HS-1793 (4) is a derivative of resveratrol that has an effect on immune cells through changing lymphocyte proliferation and the Treg cell population in FM3A breast tumor-bearing mice. It was found to promote the activity of concanavalin A-induced lymphocytes in these mice. HS-1793 has also been found to cause changes in the subset of tumor-infiltrating T cells including the CD25+ cells, in a dose-dependent manner (33).

Vanillic acid (5) is an aromatic phenolic compound produced by several different plants, such as vanilla beans. It has been found to be of benefit in different pathological conditions due to its antioxidant and antibiotic effects. This phenolic compound exhibited anti-tumor properties in mouse models with 4 T1 breast tumors, with phagocytosis and apoptosis-induction occurring via the promotion of macrophage polarization to the M1 phenotype through IL-6R/Janus kinase (JAK) signaling (34). XK-81 (6) is a novel bromophenol obtained from *Leathesia nana* that was found to have an immunotherapeutic effect on BC cell lines. It elevated the number of CD8+ T cells and NKs and modulated the ratio of M1/M2 macrophage in tumor tissues. This was combined with an elevation of immune-related cytokines, including IL-12, TNF-α, and IL-1β in a macrophage cell line (35).

### 5.2 Terpenes

Ursolic acid (7) is triterpenoid compound that exists in different fruit and vegetables and is known for its poor solubility. Research has been conducted to develop liposome-loaded ursolic acid for BC immunotherapy. It has been found to modulate CD25+ forkhead box P3 (Foxp3+) T cells via the inhibition of signal transducers and activators of the transcription (STAT)5 phosphorylation and IL-10 secretion. It also reduced MDSC population and Tregs within tumor tissue, resulting in the correction of immunosuppressive conditions generated by the TME and the inhibition of tumor growth (36). Anemoside A3 (8), from the root of *Pulsatilla chinensis*, is a triterpenoid glycoside that suppresses progression of TNBC tumors via shifting of the M2-TAM to the M1-TAM phenotype and inhibiting TAM-TNBC crosstalk (37).

Oridonin (9) is a diterpenoid with anti-inflammatory and antitumor activities. It is obtained from the plant *Rabdosia rubescens* and is used in Chinese herbal medicine. This molecule modulates Treg differentiation both *in vitro* and *in vivo*, leading to the attenuation of Treg immunosuppressive ability. This mechanism depends on the reduction of TGF-β receptor expression (38). Crassolide (10) is a natural marine product belonging to the class of cembranoid diterpenes, and is produced by Formosan soft coral, *Lobophytum michaelae*. Tsai and colleagues investigated its potential immunogenic effects and found that crassolide induced immunogenic cancer cell death. It also reduced the expression of CD24 on the surface of cancer cells and blocked mitogen-activated protein kinase (MAPK) 14 activation and STAT activity (39). Another diterpene, triptolide (11), is derived from the vine *Tripterygium wilfordii* and is used in traditional Chinese medicine as an immunosuppressant in autoimmune diseases and inflammatory conditions (78). Research has shown the ability of triptolide to act as a controller to promote cancer cell-reactive immune responses via the suppression of interferon-γ-induced PD-L1 surface expression leading to down-regulation of the PD-1/PD-L1 pathway (40).

### 5.3 Flavonoids

Naringenin (12) is a flavanone that exists in citrus fruits and has immunomodulatory, antioxidant, antidiabetic, and hypolipidemic properties. Naringenin has been shown to decrease the infiltration of MDSCs and Treg cells in breast cancer cell lines, and to upregulate IL-2 and INF-γ -releasing T cells in spleen and lung tissue, demonstrating its use as an immunomodulatory agent. Qin’s group revealed anticancer activity in tested animal model with an elevation of IL-2 and IFN-γ expressing T cells (41). Other research found that naringenin inhibited the transformation of lymphatic T cells into Tregs, which prevented *in vivo* pulmonary metastasis triggered by BC through the lowering of TGF-β1 production via the protein kinase C signaling pathway (42). Furthermore, a combination of cryptotanshinone and naringenin (13) caused a switch in immune response towards Th1 cells, thereby enhancing their activities and modulating Treg cell activity through JAK2/STAT 3 pathway in BC (43). Another flavonoid, apigenin (14), which is found in fruit and vegetables such as onions and oranges, has been found to boost immune system functioning. Apigenin has shown inhibitory effects on interferon-γ-induced PD-L1 expression as well as interferon-γ mediated STAT1 activation. One study investigated the immunogenic properties of apigenin and found that it intensified the anti-tumor immune response by increasing the proliferation of T cells (44). Salvigenin (15) is a flavonoid obtained from *Salvia miltiorrhiza* known to have cytotoxic and immunomodulatory properties. Its activity, in conjunction with the modulation of cytokine production of primed immune cells, was demonstrated by Noori’s group, who found a significant rise in anti-cancer immunity and a reduction of tumor tissues in a BC mouse model. *In vivo* results showed the reduction of IL-4 and elevation of IFN-c in the models, accompanied by suppression of Foxp3+ Treg cells (45). Another flavonoid, myricetin (16), found in tea and in berry plants, showed significant inhibitory effects on PD-L1 in IFN-γ-treated MDA-MB-231 BC cells (46).

Sativan (17) is an isoflavane produced by *Spatholobus suberectus*, another plant which is used as a remedy in traditional Chinese medicine. Peng and colleagues revealed that treatment with sativan resulted in the downregulation of the expression of PD-L1 and epithelial-to-mesenchymal transition by up-regulating miR-200c. In addition to the suppression of PD-L1, N-cadherin, snail and vimentin levels decreased, indicating the inhibition of tumor migration and invasion (47). Hesperidin (18) is classified as a flavanone glycoside and has various pharmacological effects on cardiovascular, neurological, and psychiatric conditions as well as on cancer. It is found naturally in citrus fruits. Kongtawelert and colleagues showed the suppressive effects of hesperidin on the mRNA and protein of PD-L1 in the MDA-MB-231 cell line, via inhibition of protein kinase B (Akt) and nuclear factor kappa (NF-κB) signaling (63). Sulaiman et al. investigated the properties of hesperidin via a nanoformulation of hesperidin loaded on gold nanoparticles. The results showed that the nanoformulation stimulated macrophage activity in Ehrlich ascites tumor cell-bearing mice (79).

### 5.4 Alkaloids

Berberine (19) is an alkaloid that is naturally present in a variety of plants, including barberry and oregon grape. It has various pharmacological properties, including anti-inflammatory, analgesic, hypolipidemic and antimicrobial activity. Upon exposure of BC 4T1 tumor-bearing mice to berberine, all immune-cell marker levels was significantly reduced except for CD8, and inflammatory markers were down-regulated. Furthermore, the infiltration of NKs was elevated in the treated group, revealing the immunogenic effect of berberine in BC (**Table 1**) (49). 3,3′-Diindolylmethane (20) is another natural alkaloid that has been investigated for its anti-cancer effects. It is formed during the autolytic breakdown of indole-3-carbinol, a reaction occurring in plants such as cruciferous plants. It possesses anti-tumor properties through its ability to inhibit tumor cell proliferation, suppress metastasis, and induce apoptosis of tumor cells. Moreover, Sun’s group revealed the immunogenic effect of 3,3′-Diindolylmethane as an inhibitor of MDSCs via downregulation of miR-21 levels and subsequent activation of the phosphatase and tensin homolog/PIAS3-STAT3 pathways. In addition, by raising T-cell response, it promoted the production of beneficial PD-1 antibodies and slowed tumor growth, thus indicating its potential for cancer patients undergoing anti-PD-1 treatment (50). Prodigiosin (21) is a microbial alkaloid with a red pigment that is found in the gram-negative bacterium *Serratia marcescens*. It has anti-microbial and anti-tumor properties, and is able to regulate the TME by controlling immune cells and immune checkpoints. Furthermore, it has been found to reduce the number and differentiation of Tregs, thus preventing immune tolerance and enhancing antitumor functions via inhibition of heat shock protein 90 and survivin, and activation of p53. It also suppresses tumor growth via the inhibition of M2 polarization and induction of TAM infiltration (51).

### 5.5 Peptides

Polyactin A (22) is an antibiotic belonging to the class of polymannopeptides. It can be isolated after fermentation of the buccal α-hemolytic streptococci strain. This antibiotic affects immune cells via the induction of DCs maturation from PBMCs, and the mature cells *in vitro* could initiate a potent E75 peptide-specific CD8+ T-cell response. It may have the ability to trigger the E75-specific immunologic response *in vivo* as well as *in vitro*, and has been shown to significantly increase positive rates of CD4+ and CD8+ T lymphocytes (52). Enniatin A (23) is a cyclohexadepsipeptide obtained from the Fusarium species of fungi, with the ability to reprogram the TME. It has been shown to trigger immunogenic cell death in TNBC syngeneic mice. Moreover, it can reduce PD-L1 levels and promote CD8+ T cell-dependent antitumor activity by activating the chemokine-related receptor CX3C motif chemokine receptor 1 pathway (53).

### 5.6 Polysaccharides

Fucoidans (24), are sulfated polysaccharides that can be extracted from brown algae seaweeds such as *Cladosiphon okamuranus*. They have a range of properties that include antioxidant, immunomodulatory, and anti-cancer activity. In addition to their ability to induce cell cycle arrest and apoptotic death of BC cells, fucoidans have immuno-potentiating effects in immune cells. Moreover, they can modulate the activity of adaptive and innate immune responses via the potentiation of T cells, macrophages, DCs and NKs. A study involving co-culturing co- CD8+ T cells and human breast cancer cells (MCF-7) revealed that CD8+ T cells number and IFN-γ increased more in the fucoidan-treated group. In another study, by Jin et al., similarly immunogenic effects were found to occur through the promotion of both CD4+ and CD8+ T cell responses via the elevation of immunomodulatory mediators of Th1 and CD8+ T cells (IFN-γ and TNF-α). Fucoidans also activate the maturation of DCs, either by raising levels of CD40, CD86, and MHC-I and -II surface molecules or increasing cytokines such as IL-12 and TNF-α (70). Nanoformulations of fucoidan with doxorubicin (25) combine the cytotoxic effects of doxorubicin with the ability of fucoidans to moderate the tumor microenvironment by raising the immune response of Th1 and switching M2 TAM to the M1 TAM phenotype (55). Different formulations composed of oligo-fucoidan and Olaparib (26) were found to synergistically suppress PD-L1, resulting in repression of the oncogenic IL-6/p-epidermal growth factor receptor/PD-L1 pathway. Moreover, it decreased subpopulations of CD44/CD24, suppressed M2 macrophage intrusiveness and repolarized M2 to the M1-like (CD80high and CD86high) phenotypes and induced immunoactivity and antitumoral M1 macrophages (56).

Lentinan (27) is a polysaccharide obtained from the edible mushroom *Lentinus edodes*, which has antitumor and immuno-stimulating properties. It is able to enhance immune function in the treatment of BC, as demonstrated by Guan’s group. The immunohistochemical findings showed that it reduced the mRNA expression of marker genes related to M2-type macrophage. It also suppressed M2 macrophage polarization induced by IL-4 cytokine. It activated the JAK/STAT signaling pathway, as shown by molecular docking, western blotting and siRNA transfection experiments (57). Polysaccharides from *Tetrastigma hemsleyanum* (SYQ) (28) have been found to induce the polarization of M2 to anti-tumor M1 phenotypes. This causes promotion of the macrophage polarization leading to the inhibition of BC cell proliferation (58). Polysaccharides (29) from *Ganoderma lucidum* are known to boost the immune system. Polysaccharide fractions from the plant are reported by Zhao and colleagues to activate macrophages significantly, leading to inhibition of BC cells (80). Moreover, other studies have shown the ability of polysaccharides to promote the function of several immune cells such as APCs and mononuclear phagocytes, as well as increasing humoral and cellular immunity. The latter process includes the production of CTLs and activated macrophages (59).

### 5.7 Saponins

The steroidal saponin taccaoside A (30) is one of the principal phytochemicals in many herbs used in traditional Chinese medicine, and is known for its anti-cancer activity. It was found to exhibit significant activity against BC cells by increasing granzyme B through improving the signaling of the T lymphocyte mammalian target of rapamycin 1 (mTOR1)-Blimp-1, providing *in vivo* evidence of anti-tumor efficacy (60). Ginsenosides (31) belong to the class of saponins and are known for neuroprotective and anti-inflammatory properties. The main ginsenosides are obtained from the root of *Panax ginseng*, one of these being ginsenoside Rg3, extracted from Korean ginseng, which has the ability to induce apoptosis, enhance the activity of NKs and inhibit the NF-κB signaling pathway in BC model (81). Due to its insolubility in water and poor solubility in the intestine, nanoformulations of Rg3 with doxorubicin have been designed, in which Rg3 raises infiltration levels of memory T cells in the tumor microenvironment while the doxorubicin promotes immunogenic cancer cell death (61).

### 5.8 Miscellaneous

Eribulin mesylate (32) is a natural marine product extracted from the Japanese marine sponge *Halichondria okadai* which has been approved for use in metastatic cancer. Its immunomodulatory effect was investigated by Goto et al. in conjunction with locally advanced or metastatic breast cancer. Tumor biopsies from patients receiving eribulin treatment were collected and analyzed for the expression of immune markers including PD-L1, PD-L2, CD8 and the Treg marker FOXP3. Results showed a significant reduction of immunosuppressive drivers PD-L1 and FOXP3, leading to reduced immunosuppression in the TME (62). Sesamin (33) is a lignin extracted from the oil of *Sesamum indicum*, which is recognized for antioxidant and anti-inflammatory activities. Kongtawelert’s group revealed that sesamin downregulated PD-L1 expression in both mRNA and protein in MDA-MB231 breast cancer cells. This was mediated by NF-κB and Akt, and also suppressed extracellular signal-regulated kinase (ERK) and JAK/STAT signaling activity (48).

Artemisinin (34) is sesquiterpene lactone obtained from the plant *Artemisia annua* that is used as an anti-malarial drug. Cao and colleagues explored its ability to suppress BC growth via its immunomodulatory activity. They found that artemisinin boosted T cell functioning, blocked the immunosuppressive effects of Tregs and MDSCs, and allowed CD4+ IFN-γ+ T cells and cytotoxic T cells to thrive, all of which hindered tumor growth *in vivo* (64). Oleuropein (35) is a glycosylated seco-iridoid, a type of phenolic bitter compound, extracted from *Olea europaea* L. It is known for its anti-inflammatory, anti-cancer antioxidant, neuroprotective, and anti-atherogenic properties. Hamed and colleagues revealed that oleuropein controls the miR-194/XIST/PD-L1 loop in TNBC, thus making it a promising nutritional epigenetic agent in cancer immunotherapy (65). Salinomycin (36) is an antibiotic obtained from the bacterial species *Streptomyces albus* that has been investigated for its anti-tumor activity in BC. It was found to suppress activation of the JAK/STAT pathway by IFN-γ and inhibit IFN-γ-induced activation of the NF-κB pathway by inhibiting IκB degradation and NF-κB phosphorylation. It also inhibited indoleamine 2,3 dioxygenase enzymatic activity, as shown by molecular docking, where salinomycin demonstrated nucleophilic attack in the catalytic domain of indoleamine 2,3 dioxygenase. In tumor tissue, *in vivo* research found that salinomycin activated cisplatin’s anti-tumor properties and appeared to boost T cell production when co-cultured with BC cells treated with IFN-γ (66).

Metformin (37) is a known hypoglycemic drug that can be extracted from *Galega officinalis*. Results reported by Cha’s group show that metformin has the potential to lower PD-L1 in breast cancer by activating protein kinases via AMP-activated protein kinase (AMPK). Blocking PD-L1 signaling enhances CTLs activity against tumor cells (67). Alpha-tocopheryl succinate (α-TOS) is one of the forms of vitamin E that is an effective anti-tumor agent. A nanoparticle delivery system was designed for α-TOS (38) which aimed to boost anticancer immunity through the suppression of IFN-γ-induced PD-L1 expression. It also enhanced tumor elimination via the modulation of cytotoxic lymphocyte infiltration into the TME (68). Neo-tanshinlactone (39) is a planar natural molecule having four rings. It is obtained from *Salvia miltiorrhiza* Bunge and is known as an ICI and as PD-1/PD-L1 interaction inhibitor. Zhang’s group investigated the effect of different analogues of neo-tanshinlactone against TNBC. MZ58 (40) proved to be the best candidate in a subcutaneous transplantation tumor model as it showed less cytotoxicity toward T cells, activated CD8+ T cells, and reduced T cell exhaustion (69).

### 5.9 Combinations of natural compounds

In some cases, the effect of single compound can be strengthened by combining it with other phytochemicals. This phenomenon has been investigated in studies aimed at remodeling BC cells using the synergistic effects created in combining active chemical constituents (**Table 1**). For example, a combination of curcumin, resveratrol, and quercetin (RCQ) (41) was designed to manipulate the multi-layered interactions of cells and signaling pathways in a novel approach to phyto-immunotherapy in BC. The RCQ combination has been shown to remodel antitumor immunity in 4T1 breast cancer-bearing mice by shifting the immune balance toward an immune activation state by reversing the superiority of immunosuppressive infiltrating cells in the TME. This is achieved by the inhibition of the development of TILs into immunosuppressive cells, including TAMs cells to M2 TAMs and TANs to the N2 TANs. It also enhanced the T cells accumulation and decreased the recruitment of macrophages and neutrophils in the TME (54).

### 5.10 Extracts

Ethanolic extracts of *Cordyceps militaris* (42) have shown immunogenic effects in stimulating the proliferation of tumor-specific T cells without inhibiting DCs functioning and T cell proliferation (82). Yang et al. isolated *C. militaris* immunoregulatory protein that suppresses the proliferation of 4T1 breast cancer cells. The immunoregulatory protein elevated the mRNA levels of cytokines IL-6, IL-1β and TNF-α in peritoneal macrophages (71). In addition, a traditional Chinese formula (XIAOPI) (43), composed of 10 plants, has shown the ability to reprogram the TME by decreasing the polarization and proliferation of M2-type macrophages. Similarly, other herbal extracts from *Cordyceps sinensis*, *Taraxacum mongolicum*, and protein-bound polysaccharides (from the *Coriolus versicolor* fungus) (44) suppressed progression of TNBC via shifting the M2-TAM to the M1-TAM phenotype, and inhibiting TAM-TNBC-talk (37). Regarding protein-bound polysaccharides from *C. versicolor* (45), several clinical studies have been conducted showing their significant immunological and oncological activity, with overall improvement of prognosis for BC patients. The mechanism of action involves a Th1 adaptive immune response and modulation of immunosuppressive TME via activation of DCs (72). A polysaccharide fraction obtained from *Astragalus membranaceus* (46) has been shown to increase immune response. This effect is linked to the inflammatory immune response at the tumor site. Detailed investigation of the mechanism showed reduction of the expression of PD-L1 via the Akt/mTOR/ribosomal protein S6 kinase beta-1 pathway (73).

The fruit extract of *Diospyros peregrina* (47) has been evaluated for its immunogenic effect on BC models. Findings show that the extract controlled and combatted BC, and suppressed the expression of Foxp3+Treg cells within tumor tissue. This was reflected in immune cell and cytokine activity, as the release of Th2 cytokines was reduced, including IL-10 and IL-4, and the release of Th1 cytokines, including IL-12 and IFN-γ, was elevated. This was accompanied by elevation of the activity of T-box transcription factor TBX21and the suppression of the expression of transcription factor FOXP3 and GATA binding protein 3 (74). *Sarcodon imbricatus* (48) as an aqueous extract, showed inhibitory effects on the growth, migration, and invasion capacity of BC cells. It decreased the expression of PD-L1 in BC models and elevated IL-2, IL-6, TNF-α, and NKs activity (75). Yang and colleagues prepared an ethyl acetate fraction from a mixture of *Hedyotis diffusa* and *Scutellaria barbata* (49) revealing their ability to reduce the expression of PD-L1, β-catenin, and cyclin D1, causing inactivation of MAPK and Akt signaling pathways (76).

Badawy et al. investigated the properties of camel milk (50) and its exosomes nanoparticles. *In vitro* as well as *in vivo* tests using oral and local injection, found that camel milk reduced breast tumor progression via several different mechanisms, including apoptosis induction, oxidative stress inhibition, suppression of several types of gene-related inflammation (IL1b, NF-κB), angiogenesis (vascular endothelial growth factor) and metastasis (matrix metalloproteinase-9, intercellular adhesion molecule 1). These anti-tumor effects were accompanied by higher immune response, evidenced by higher numbers of CD+4, CD+8, and NKs (77).

## 6 Perspectives and conclusion

This review demonstrates the potential role of natural products as immunotherapeutic treatments in BC. The risks and side-effects of modern cancer chemotherapy are well-known, and research into less debilitating treatments such as immunotherapy is increasing steadily. Plant, animal, microbial, and marine organisms continue to provide sources of structurally diverse and biologically active compounds that are able to regulate the human body’s immune response. The findings of this review reveal the wide range of different immune cells, cytokines, and signaling pathways that can be modified and regulated to fight cancer tumors. Natural compounds can suppress immunosuppressive cells such as Tregs and MDSCs can significantly lower treatment response, as can immunosuppressive mediators such as IL-10 and TGF-β. Active compounds derived from natural products have been shown to effectively stimulate immune cells such as CD+8 cells, NKs, DCs and TAMs, which then block immune suppression in the TME. Active compounds also promote the secretion of anti-tumor immune factors (IFN-γ, TNF-α, IL-1). Research has shown that some can inhibit signaling pathways such as NF-κB, JAK-STAT, MAPK and Akt/mTOR, and stimulate immunogenic cancer cell death. Others have been shown to inhibit immune checkpoints such as PD-L1 in both *in vitro* and *in vivo* studies.

Immunomodulatory natural compounds can be delivered in a variety of different forms, such as extracts, isolated entities, synthetic derivatives, nanoformulations, and compound combinations. For example, extracts of *Coriolus versicolor, Cordyceps militaris* and *Astragalus membranaceus* successfully stimulated significant immunological and oncological activity with overall improvements in the prognosis for BC. Different classes showing chemical diversity, such as flavonoids, alkaloids, terpenes, peptides and polysaccharides exert modulatory effects on immune cells, immune cytokines and immune checkpoints. RCQ is an example of the successful combination of compounds, with its synergistic activity leading to inhibition of immunosuppressive cells in the TME and restoration of immune balance and immune activation to fight BC growth. Nanoformulations have also been successively designed and developed to combat immunosuppressive TMEs, an example being curcumin-loaded polymeric nanoparticles, which, once injected, triggers immunogenic cell death of cancer cells in BC models. The combination of immunotherapeutic compounds with chemotherapy shows outstanding effects on immune tolerance in BC. Furthermore, a nanoformulation of fucoidan combined with the chemotherapeutic drug doxorubicin significantly enhanced doxorubicin’s effects and caused manipulation of the immune landscape of the tumor to increase immune cell response against cancer cells.

Bioactive compounds have been shown, in *in vivo* and *in vitro* experiments, to have a positive effect on the immune system and its ability to fight cancer cells. This has been especially valuable when using TNBC and 4T1 BC mouse models, where natural products can be evaluated in terms of their immunotherapeutic activity on the TME in BC progression. Recent clinical studies have shown that natural compounds can provide adjunctive immune support in TNBC patients. For example, protein-bound polysaccharides from *C. versicolor* have demonstrated significant immunological and oncological activity, resulting in overall improvements in BC prognosis. Unfortunately, most natural active compounds are not readily translatable into clinical trials where pharmacokinetics, stereochemistry, and bioavailability are all considerations for efficient drug delivery. In addition, current restrictions applied to clinical trials may affect the extent to which some natural compounds can be used in drug combinations. As an example, the bioactive alkaloid matrine, found in plants of the Sophora species (e.g. *Sophora flavescens*), has been shown to cause autophagic and apoptotic death in BC cell lines. Despite demonstrating significant ability to regress tumors and suppress metastasis in TNBC mouse models, matrine has only been studied in a few clinical trials in China, and there has been no definitive positive consensus in the findings. In other clinical trials, BC patients were treated with aqueous extracts of *Sophora flavescens* and *Smila glabra* as part of a combination therapy alongside conventional chemotherapy. However, some of these studies revealed a better clinical response compared to control group than others while others showed no response. Nevertheless, the studies reported improvements in quality of life resulting from a decrease in chemotherapy toxicity.

Compared to conventional drugs, natural products exhibit several advantages; wide availability, fewer and less severe side-effects, diverse pharmacological and chemical properties including immunomodulation, apoptotic induction, proliferation suppression, and metastasis inhibition, which all together contribute to cancer cell death. These factors indicate the potential for naturally-occurring compounds to play a significant role in tumor immunotherapy. However, there are a few issues that need to be addressed. For example, in order to identify the most effective natural compounds, we need more comprehensive and in-depth research into immune-system signaling pathways with respect to tumor immunotherapy. Also, determining the range of safe dose is challenging, since potential toxicity to vital organs such as liver and kidneys needs to be taken into account. In addition, there are research challenges relating to variations in the TME and in tumor heterogeneity, as well as difficulties in elucidating the molecular mechanisms and identification of targets relevant to tumor immunity. Also, the bioavailability of some natural products could limit their applications *in vivo*, as repeated doses are required, leading to increased risk of toxicity. Using advanced techniques to overcome these challenges could improve the likelihood of successful cancer immunotherapy natural drug development. Natural products can be incorporated in unique drug delivery systems, computerized design techniques, and metabolomics. These could lead to fruitful future strategies for clinical breast cancer treatments.

## Author contributions

AA: Conceptualization, Data curation, Investigation, Methodology, Resources, Software, Writing – original draft, Writing – review & editing.
